# A pivotal registration phase III, multicenter, randomized tuberculosis controlled trial: design issues and lessons learnt from the Gatifloxacin for TB (OFLOTUB) project

**DOI:** 10.1186/1745-6215-13-61

**Published:** 2012-05-18

**Authors:** Corinne SC Merle, Charalambos Sismanidis, Oumou Bah Sow, Martin Gninafon, John Horton, Olivier Lapujade, Mame Bocar Lo, Denis A Mitchinson, Christian Perronne, Francoise Portaels, Joseph Odhiambo, Piero Olliaro, Roxana Rustomjee, Christian Lienhardt, Katherine Fielding

**Affiliations:** 1 Faculty of Epidemiology and Population Health, Tropical Epidemiological Group, London School of Hygiene and Tropical Medicine, Keppel Street, London, WC1E 7HT, UK; 2 Service de pneumo phtisiologie, Hopital Ignace Deen, Conakry, Guinea; 3 Centre National Hospitalier de Pneumo-Phtisiologie, Cotonou, Benin; 4 Tropical Projects, Hitchin, UK; 5 WHO/TDR, 20 av Appia, CH-1211, Geneva 27, Switzerland; 6 Programme National de Lutte contre la Tuberculose, BP 5899, Dakar, Fann, Sénégal; 7 Centre for Infections, St George’s, University of London, SW17 0RE, London, UK; 8 Infectious Diseases Department, Raymond Poincaré Hospital, Assistance Publique – Hôpitaux de Paris, Université de Versailles, Saint Quentin, France; 9 Françoise Portaels, Department of Microbiology, Tropical Institute of Medicine of Antwerp, Antwerp, Belgium; 10 Kenya Medical Research Institute, Off Mbaghathi Road, P.O. Box 47855, Nairobi, Kenya; 11 WHO/TDR, 20 av Appia, CH-1211, Geneva 27, Switzerland; 12 Medical Research Council, Unit for Clinical and Biomedical TB Research, 491 Ridge Road, P.O. Box 70380, Overport, 4067, South Africa; 13 WHO Stop TB partnership 213, rue La Fayette, Geneva, Switzerland

**Keywords:** Randomized controlled trial, Tuberculosis treatment, Non-inferiority design, Multicenter, Design issues, Phase III, Gatifloxacin

## Abstract

**Background:**

There have been no major advances in tuberculosis (TB) drug development since the first East African/British Medical Research Council short course chemotherapy trial 35 years ago. Since then, the landscape for conducting TB clinical trials has profoundly changed with the emergence of HIV infection, the spread of resistant TB bacilli strains, recent advances in mycobacteriological capacity, and drug discovery. As a consequence questions have arisen on the most appropriate approach to design and conduct current TB trials. To highlight key issues discussed: Is a superiority, equivalence, or non-inferiority design most appropriate? What should be the primary efficacy outcome? How to consider re-infections in the definition of the outcome? What is the optimal length of patient follow-up? Is blinding appropriate when treatment duration in test arm is shorter? What are the appropriate assumptions for sample size calculation?

**Methods:**

Various drugs are currently in the development pipeline. We are presenting in this paper the design of the most recently completed phase III TB trial, the OFLOTUB project, which is the pivotal trial of a registration portfolio for a gatifloxacin-containing TB regimen. It is a randomized, open-label, multicenter, controlled trial aiming to evaluate the efficacy and safety of a gatifloxacin-containing 4-month regimen (trial registration: ClinicalTrial.gov database: NCT00216385).

**Results:**

In the light of the recent scientific and regulatory discussions, we discuss some of the design issues in TB clinical trials and more specifically the reasons that guided our choices, in order to best answer the trial objectives, while at the same time satisfying regulatory authority requirements.

**Conclusion:**

When shortening TB treatment, we are advocating for a non-inferiority, non-blinded design, with a composite unfavorable endpoint assessed 12 months post treatment completion, and added trial procedures specifically aiming to: (1) minimize endpoint unavailability; and (2) distinguish between relapse and re-infection.

## Background

Tuberculosis (TB) has long been neglected as a public health problem and remains, with HIV/AIDS, one of the most important cause of death from a single infectious agent among adults in developing countries [1,2]. There were an estimated 8.8 million new TB cases in 2010 [3]. Drug susceptible TB is currently treated with a 6-month course regimen: 2 months intensive phase of daily isoniazid, rifampicin, pyrazinamide, and ethambutol followed by 4 months continuous phase of daily isoniazid and rifampicin [4]. While generally effective with proper compliance, in practice patients may fail to adhere to treatment at any time and default. The consequences may be treatment failure for the individual patient but also, for the general population, the risk of the emergence and spread of resistance when the inadequate treatment selected for drug-resistant bacteria. A shorter duration of treatment is expected to provide improved patient adherence and better treatment outcome.

Shortening the duration of treatment has been recognized by both the World Health Organization (WHO) Stop TB and the Global Alliance for TB Drug Development (GATB) as a major target for the improvement of TB control worldwide [3]. Furthermore, at the time of developing this study new compounds for TB treatment were still at the early stage of clinical development, and investigation of the effect of existing drugs was a priority. The fluoroquinolones have proven to be useful in the management of multidrug-resistant TB, and have been proposed for shortening first-line treatment of pan-susceptible TB [5-7]. Evidence for the potential shortening of treatment using fluoroquinolones was first provided in observational studies where ofloxacin replaced ethambutol in India [8]. The third generation fluoroquinolones, gatifloxacin and moxifloxacin, have been shown to have better bactericidal activity than ofloxacin *in vitro* and *in vivo*[6], and confirmed by a serial sputum colony counts phase II study [9]. It was therefore considered by authorities in the field (including WHO Stop TB, GATB, and WHO/TDR) that proper investigation of the third generation fluoroquinolones was justified, and should be pursued. The choice of gatifloxacin as the molecule under investigation in the OFLOTUB trial was based on its bactericidal activity profile and its generic status.

In this paper we present issues that arose in the design of the trial, with emphasis on salient methodological aspects that have been source of various discussions within the team and with regulatory authorities.

## Methods

### Overview of OFLOTUB

OFLOTUB is a pivotal phase III, randomized, open-label, parallel group, multicenter trial included in the registration portfolio for a gatifloxacin-containing TB regimen. The objective of the trial is to evaluate the efficacy and safety of a gatifloxacin-containing 4-month regimen (test regimen) in the treatment of pulmonary TB in comparison with the standard WHO-recommended 6-month regimen (reference regimen) (Table 1). Subjects were randomized in a ratio of 1:1 to receive either the test or standard regimen. Randomization was stratified by country and conducted using sealed envelopes. Each patient is followed for 24 months after the end of treatment. The trial has been designed to comply with GCP/ICH guidelines (details on the implementation of these for our trial will follow in a future publication).

**Table 1:** Treatment regimens

**Test regimen**	**Intensive phase (2 months)**	**< 50 kg (6 days/week)**	**≥ 50 kg (6 days/week)**	**Continuation phase (2 months)**	**< 50 kg (6 days/week)**	**≥ 50 kg (6 days/week)**
	Combination tablet	3 tablets	4 tablets	Combination tablet	3 tablets	4 tablets
	Isoniazid 75 mg			Isoniazid 75 mg		
	Rifampicin 150 mg			Rifampicin 150 mg		
	Pyrazinamide 400 mg					
	Gatifloxacin 400 mg	1 tablet	1 tablet	Gatifloxacin 400 mg	1 tablet	1 tablet
						
**Control regimen**	**Intensive phase (2 months)**	**< 50 kg (6 days/week)**	**≥ 50 kg (6 days/week)**	**Continuation phase (4 months)**	**< 50 kg (6 days/week)**	**≥ 50 kg (6 days/week)**
	Combination tablet	3 tablets	4 tablets	Combination tablet	3 tablets	4 tablets
	Isoniazid 75 mg			Isoniazid 75 mg		
	Rifampicin 150 mg			Rifampicin 150 mg		
	Pyrazinamide 400 mg					
	Ethambutol 275 mg					

### Trial setting

Patients were recruited in five sites in Africa: Benin (Cotonou, National TB program); Guinea (Conakry, National TB program); Kenya (Nairobi, KEMRI); Senegal (Dakar, National TB program); and South Africa (Durban, MRC). All trial products were provided by Lupin Pharmaceuticals, Mumbai, India. Thammasat University, Bangkok, Thailand is responsible for the central data management of the trial in collaboration with the local data-management teams based in each of the recruitment sites.

### Trial governance

The trial is co-sponsored by the Institut de recherche pour le developpement (IRD) and the UNICEF/UNPD/World Bank/WHO Special Program for Research and Training in Tropical Disease (TDR) and the whole drug development project is coordinated by the TDR secretariat. Two committees were initially established: A Trial Steering Committee (TSC) and a Product Development Team (PDT). The TSC monitors the conduct of the phase III trial and is composed of the Principal Investigators (PI) from each of the recruitment sites and the PIs from the other collaborating institutions: Assistance Publique - Hôpitaux de Paris, Institut de Recherche et Developpement (IRD), France; London School of Hygiene and Tropical Medicine (LSHTM); St Georges Hospital Medical School (UK); and the Institute of Tropical Medicine in Antwerp, Belgium. The PDT is composed of four independent scientists and the two co-sponsor representatives and has an over-view of the whole Gatifloxacin for TB drug development portfolio.

A Data Monitoring Committee (DMC) composed of three independent scientists monitored the safety aspects of the Phase III trial. They convened every 6 months until all patients completed treatment and 30 days of follow-up.

The trial protocol was reviewed and approved by all central and local Institutional Review Boards. The trial is registered in the ClinicalTrial.gov database with the registration number: NCT00216385.

### Recruitment of patients

Male and female patients, aged 18 to 65 years, suffering from recently diagnosed, microscopically proven, pulmonary tuberculosis, and providing informed consent for inclusion in the trial, were considered for enrolment. The full list of inclusion and exclusion criteria is presented in Table 2.

**Table 2:** Inclusion and exclusion criteria

**Inclusion criteria**	**Patients eligible for inclusion in the trial must fulfil all of the following criteria:**
	Aged 18 to 65 years (both inclusive) and weighing between 38 and 80 kg
	Recently diagnosed, microscopically proven, pulmonary tuberculosis, defined as 2 consecutively positive sputum smears, of which one must be equal or exceed grade 1
	Findings in medical history and physical examination not exceeding grade 2 according to the Division of Microbiology and Infectious Disease grading system tables (DMID)
	Voluntarily signed informed consent to participate in the trial
	Females of childbearing potential must have a confirmed negative pregnancy test at the screening visit and must employ an effective and acceptable method of birth control during the treatment
	Laboratory values that do not exceed grade 2 using the Division of Microbiology and Infectious Disease grading system (DMID) other than for glycaemia, haemoglobin, and potassium levels
	
**Exclusion criteria**	**Patients who meet any of the following criteria are not eligible for the trial:**
	Patients with a history of TB treatment within the last 3 years
	Concomitant infection requiring additional anti-infective treatment (especially antiretroviral medication)
	HIV infected patients with WHO stage 3 infection (except those presenting with only the ‘loss of weight >10% body weight’ criterion) and all patients at WHO stage 4
	A history of diabetes mellitus (DM) or non-insulin-dependent diabetes mellitus (NIDDM) requiring treatment or diet. Additionally patients who have a fasting glucose level less than 70 mg/dl (3.9 mmol/L) or above 115 mg/dl (6.4 mmol/L) at screening will be excluded
	Recreational drug abuse and alcohol abuse that, in the opinion of the investigator, could prejudice the conduct of the trial in that patient
	History of drug hypersensitivity or/and active allergic disease
	Impaired renal, hepatic, or gastric function that may, in the opinion of the investigator, interfere with drug absorption, distribution, metabolism, or elimination
	Any other findings in medical history and physical examination exceeding grade 2 in the DMID grading system tables
	Patient using the following therapies:
	Other antibiotics with known anti-TB activity (that is ofloxacin, moxifloxacin, kanamycin, and so on)
	Drugs known to prolong the QT interval (that is antiarrythmics, psychotropics (phenothiazines, tricyclics, tetracyclics), erythromycin, pentamidine, and halofantrine)
	Drugs known to give photosensitivity reactions
	Receiving oral corticosteroids for more than 2 weeks immediately prior to inclusion
	Use of antacids containing aluminium or magnesium salts or sucralfate
	Digoxin
	Drugs that are eliminated via tubular secretion (for example, probenecid, cimetidine, ranitidine)
	Pregnant or lactating women
	Patients with congenital QT interval prolongation defined as > 480 ms
	Patients with clinically significant bradycardia defined as <40 bpm
	Baseline laboratory values exceeding grade 2 using the Division of Microbiology and Infectious Disease grading system (DMID) except for haemoglobin and hypokaliaemia for which the limit values are:
	Potassium <3.0 mEq/L (>grade 1)
	Haemoglobin < 6.5 gm/dl
	Separate criteria are required for glycaemia, as listed above
	Any other finding considered by the investigator as compromising the participation of the patient in the trial
	Any condition rendering the patient unable to understand the nature, scope, and possible consequences of the trial and to provide consent
	Participation in another drug trial within the 3 months before the screening visit

### **Description of interventions (Table**1**)**

The test regimen has a four-drug 2-month intensive phase followed by a three-drug 2-month continuation phase. The reference regimen is the standard four-drug 2-month intensive phase with a two-drug 4-month continuation phase. Gatifloxacin is substituted for ethambutol in the intensive phase and is maintained for the continuation phase. Gatifloxacin is given at a dose of 400 mg per day, irrespective of body weight (1 × 400 mg once daily). The doses of isoniazid, rifampicin, pyrazinamide, and ethambutol follow WHO recommendations, as indicated in Table 1, and were provided as fixed dose combination tablets.

All trial drugs were administered orally. The administration of both regimens was supervised daily using Directly Observed Therapy (DOT). During the intensive phase, supervision was ensured by either the health center staff or a designated representative. During the continuation phase, treatment was delivered weekly or biweekly to a supervisor who ensured that daily doses are taken by the patient.

### Clinical and mycobacteriological trial procedures

The timetable of scheduled patient evaluations during and after the end of treatment is presented in Table 3. As a result of changes to the prescribing information for gatifloxacin occurring during the active phase of the study, the monitoring of blood glucose profiles of patients recruited into the trial has been intensified and comprised measurements of blood glucose at screening, 4 h, 7 and 14 days after the first drug intake, and at months 1, 2, and 3, and end of treatment. Drug safety has been closely monitored during the course of the study in compliance with ICH/GCP guidelines.

**Table 3:** Trial organizational timetable

	**Screening Treatment**								**Follow-up**										
Visit number	1	2	3	4	5	6	7	8	9	10	11	12	13	14	15	16	17	18 (G)	18 (T)
Weeks (W)	W −1	W 0	W4	W 8	W 12	W 16	W 20	W 24	W 28	W 36	W 44	W 52	W 60	W 72	W 84	W 96	W 108	W 112	W120
Demographics	X																		
Medical history	X																		
Previous treatment	X																		
Inclusion and exclusion criteria	X	X																	
Subject information Informed Consent	X																		
Physical examination	X	X	X	X	X	X	X	X	X	X	X	X	X	X	X	X	X	X^a^	X^b^
Pregnancy test	X					X^a^		X^b^											
HIV test	X																		
Laboratory tests and B/S^c^	X	X^d^	X	X	X^f^	X^a^		X^b^											
12-lead ECG	X		X	X		X^a^		X^b^											
MGIT test	X																		
Sputum smear	X			X	X^|e^	X	X	X	X	X	X	X	X	X	X	X	X	X ^A^	X^b^
Sputum culture	X			X	X^|e^	X	X	X	X	X	X	X	X	X	X	X	X	X^a^	X^b^
Indirect drug susceptibility testing	X			X		X	X	X	X	X	X	X	X	X	X	X	X	X^a^	X^b^
Chest X-ray	X					X^a^		X^b^											
Randomization		X																	
Adverse events/and events			X	X	X	X	X	X	X	X	X	X	X	X	X	X	X	X^a^	X^b^
Concomitant therapy check		X	X	X	X	X	X	X	X	X	X	X	X	X	X	X	X	X^a^	X^b^
Compliance check			X	X	X	X	X^b^	X^b^											

^a^Only for patients randomized in gati arm.

^b^Only for patients randomized in control arm.

^c^Laboratory tests include: full blood count, kalaemia, creatinin, ASAT, ALAT, amylasemia, glycemia (blood glucose is measured twice during the screening).

^d^Blood glucose levels is measured 4 h after the first dose of medication, and at fasting stage on days 8 and 15, and week 12.

^e^If sputum smears are still positive at week 8.

^f^Only blood glucose level is measured at week 12.

### Trial status

Recruitment for OFLOTUB started on June 2005 and was concluded on 31 October 2008. A total of 1,836 patients have been recruited into the trial; 316 in Benin, 452 in Guinea, 200 in Kenya, 358 in Senegal, and 510 in South Africa. In April 2011 all patients completed 24 months of follow-up after the end of treatment.

## Results and discussion of methodological issues

### Choice of a non-inferiority design

An established highly efficient treatment, that lasts 6 months, exists for pulmonary TB with associated long-term relapse rates in the region of 5% [10]. Given this, it is unlikely that a new test regimen will demonstrate superiority over the current standard regimen and therefore interest lies with showing whether a new regimen is not inferior to the standard regimen. Shortening treatment duration to less than 6 months is expected to improve adherence to treatment and help with patient management in overburdened health systems in high TB prevalence settings. It will also decrease exposure of patients to toxic drugs. These benefits are considered so essential when developing novel TB regimens that one may accept some loss of efficacy compared to the standard TB regimen for a higher effectiveness in programmatic terms. The non-inferiority design appears therefore as the best choice in current development for drug sensitive TB. A comprehensive discussion on the choice of non-inferiority design in the TB context is presented elsewhere [11]. All three current phase III trials in TB drug development investigating novel chemotherapy combinations, including quinolones, are non-inferiority trials [12-14]. OFLOTUB is designed to disprove the null hypothesis that the gatifloxacin-containing drug regimen is clinically inferior by a given the margin of non-inferiority to the standard treatment, while at the same time decreasing duration of treatment to four months. The alternative hypothesis is that the gatifloxacin-containing drug regimen is not clinically worse than (that is non-inferior to) the standard treatment.

### Implication of the non-inferiority design on the trial population to be analyzed

It is widely accepted that in non-inferiority designs there is no one single least-likely-to-be-biased population analysis. The intention-to-treat (ITT) approach aims to minimize differences by including protocol deviations and hence increases the possibility of declaring non-inferiority between arms. The per-protocol analysis is biased in that it does not include all randomized patients. In fact, both population analyses are important and concurring results are required in order for a non-inferiority trial to be conclusive [15].

Complicating matters further, in TB drug trials, a modified-ITT (MITT) population, a subset of the ITT population, is analyzed as there is a subset of patients, identifiable after randomization, who must be excluded on the basis of their *Mycobacterium tuberculosis* strain phenotype. The MITT in OFLOTUB is defined as the population of patients assigned a randomization number and dispensed trial medication on at least one occasion and excluding patients with: (1) confirmed multidrug-resistant TB on drug sensitivity testing; or (2) rifampicin resistant, invalid, or contaminated MGIT test results.

The PP population in OFLOTUB is all patients included in MITT excluding any patient missing more than two consecutive doses during the intensive phase or more than six consecutive doses (more than 1 week of medication) during the continuation phase or taking less than 80% of all doses over a 16- or 24-week period for the test and control regimens, respectively.

### Trial measures

#### Primary efficacy outcome

Over 50 years of TB clinical trials, trial endpoints have evolved as regimens and diagnosis improved. In 1947, the endpoints of the BMRC Streptomycin Trial were survival and clinical/radiological improvement [16]. Nowadays, the outcomes of interest in assessing the efficacy of new TB treatments are bacteriological and until recently, TB relapse was the main endpoint of interest. In the initial version of the OFLOTUB phase III protocol, the primary endpoint was relapse, defined as two positive cultures in two sputum collections taken consecutively at least 1 day apart during the follow-up period, restricted to patients who had demonstrated cure at the end of treatment (Table 4). For the purpose of TB treatment trials cure at the end of treatment is defined as two consecutive cultures negative at the end of treatment which differs from standard TB control programme definitions of cure. Lately, the primary outcome was changed to one of the original secondary endpoints: a composite ‘unfavourable outcome’ endpoint including failure of treatment (either at 4 or 6 months depending on arm randomized to), recurrence and other poor outcomes such as loss to follow-up during the treatment phase, death, and so on. The reason for this was primarily to ensure that all randomized patients contributed to the analysis of the trial’s primary endpoint and was following the recommendations made by the FDA on the submission of a pre-IND file. This has had an impact in the calculation of the sample size (see later) but it also forced us to define precise rules for classification of all patients to ‘favourable’ or ‘unfavourable’ categories. Table 4 gives a summary of the classification used. In some situations, the status of the patient would be classified as not-assessable. An independent endpoints committee, blinded to the trial arms, will be requested to re-classify these patients to either category. Table 5 lists all primary and secondary outcomes. Both of the other current phase III TB trials also consider a combined outcome measure, including failure to treatment and relapse, as their primary outcome of interest [12,13]. 

**Table 4:** Re-classification decisions concerning the major efficacy outcome

	**Situations**	**Classification**	**Comments/Date of event**
*Treatment phase*			
1	Two cultures + ve^a^ at the end of the treatment phase	Unfavorable	Last scheduled day of the treatment
2	One culture + ve + second culture not done or contaminated	Unfavorable	Last scheduled day of the treatment
Death			
3	Death: no information suggesting it was not related to TB	Unfavorable	Date of death occurrence
4	Death: documented evidence that it was not related to TB (for example, car accident)	Unfavorable	Date of death occurrence
Withdrawals (other than death and before the end of treatment)			
5	SAE^b^ for which treatment is stopped	Unfavorable	Date when TB treatment stopped
6	Pregnancy (so treatment is stopped)	Not assessable	Date when TB treatment stopped
7	Patient withdrawal of consent	Unfavorable	Date of withdrawal
			
*Follow-up phase*^c^			
1	Culture -ve + data available for all visits or data available for last follow-up visit	Favorable	Date of last culture -ve
2	Two cultures + ve (at least 1 day apart) during follow-up and organism id is Mtb	Unfavorable	Date of the first sampling with culture + ve
3	One culture + ve and organism id is Mtb + second culture not done or contaminated	Unfavorable	Date of the sampling with the culture + ve
4	Death and one culture + ve based on most recent culture result and organism id is Mtb	Unfavorable	Date of the sampling with culture + ve
5	Death and culture -ve based on most recent culture result (or contaminated)	Not assessable or Unfavorable	Endpoint committee reviews available data
Withdrawals (other than death):			
6	Patient withdrawal of consent and one culture + ve based on most recent culture result	Unfavorable	Date of the sampling with culture + ve
7	Patient withdrawal of consent and culture -ve based on most recent culture result (or contaminated)	Non-assessable for the period after being withdrawn	Favorable till the date of last culture -ve/Non-assessable the day after the last visit
8	Lost during follow-up/moved away from area and one culture + ve based on most recent culture result	Unfavorable	Date of the sampling with culture + ve
9	Lost during follow-up/moved away from area and culture -ve based on most recent culture result (or contaminated)	Non-assessable for the period after being withdrawn	Favorable till the date of last culture -ve Non-assessable the day after the last visit

^a^Positive.

^b^Serious adverse event.

^c^Defined among those patients classified as cured at the end of the treatment phase. Cure is defined as two consecutive negative cultures.

**Table 5:** Primary and secondary objectives

**Efficacy outcomes**	
*Primary efficacy outcome*	Percentage of unfavourable outcomes by 24 months following the end of treatment
*Secondary efficacy outcomes*	
	-Percentage of unfavourable outcomes by 18 months post randomization
	-Percentage of recurrences by 24 months following the end of treatment. This is based on those individuals who have achieved cure at the end of the treatment
	-Time to recurrence, defined from the date of treatment cure to the date of relapse
	-Percentage of patients with sputum culture conversion at 8 weeks
	-Percentage of patients with sputum smear negativity at 8 weeks
	-Percentage of patients cured by the end of treatment
	-Time to a composite (unsatisfactory) endpoint of treatment failure or relapse
**Safety outcomes**	
*Primary safety outcome*	Percentage of adverse events
*Secondary safety outcome*	The distribution of type and grading of adverse (based on DMID tables)

#### Bacteriological diagnosis

False-positive cultures can result from misidentification of the strain, laboratory cross-contamination, or clerical error in reporting. Burman *et al.* found that false-positive culture results were detected in 13 of 14 DNA fingerprinting studies that evaluated more than 100 patients, with a median false-positive rate of 3.1% (interquartile range 2.2-10.5%) [17]. In the BMRC trials, half of the isolated positive cultures (IPC) were seen to be the results of laboratory cross contamination [18]. The risk of misclassification is far from negligible and can be minimized if two positive specimens are available to support the diagnosis. Therefore, we choose to base the diagnosis of failure at end of treatment or recurrence following documented cure on two cultures results instead of just one.

#### Recurrence, relapse, and re-infection

Relapse (reactivation of the original infection) and re-infection (infection with a new TB strain) are two different events leading to the same clinical outcome which defines, in part, the primary composite outcome of our trial. An effective regimen should be able to prevent relapse but have no effect on re-infection. In a context of a significant burden of both TB and HIV, such as in our site in Durban, the proportion of recurrences due to re-infection can be high [19,20]. Even if these will be balanced across arms in a randomized trial, they could dilute the real regimen effect which is primarily assessed by relapses. For the same reason ITT analysis might be biased in non-inferiority trials, considering patients with re-infection as recurrence might tend to minimize differences between study arms thereby increasing the possibility of declaring non-inferiority if the percentage of re-infections is important compared to the percentage of relapses. This can have a deleterious effect in the context of the non-inferiority design by forcing non-inferiority. In order to account for this, we are differentiating relapse from re-infection using a molecular method (MIRU-VNTR) by comparing baseline and recurrent strains [21]. However, to allow for direct comparison with previous trials, recurrence will also be considered in our analysis.

#### Duration of the follow-up

We initially took the conservative approach of following up patients to 24 months after the end of treatment. This decision has been highly driven by the desire for comparability of our results with the past British Medical Research Council (BMRC) short course chemotherapy trials [22]. However, the majority of recurrences occur in the first year post treatment and there is an inherent difficulty in maintaining patients in trials for long durations of follow-up [23]. Therefore, a shorter follow-up may be sufficient to demonstrate non-inferiority of the test regimen while simultaneously decreasing the risk of loss of trial power due to patients lost to follow-up. How long is long enough? This is a matter of debate. Some propose 6 months after the end of treatment [23]. In the light of the results of the BMRC trials [22], 1 year of follow-up seems to capture most of the relapse cases. Therefore, in addition to the endpoint analysis 24 months after completion of treatment, we are also assessing our endpoints at 18 months post randomization (that is 12 and 14 months after completion of the treatment for the control and test arms, respectively). This will allow for comparison of our results with those of the other two current phase III trials where patients are followed up for 18 months after randomization.

### Implications of a non-inferiority design on major outcome measurement

Completeness of outcome data is of critical importance for non-inferiority trials. This is to ensure non-inferiority is actually established based on true regimen performance rather than on missing data, associated loss of power, and thus decrease the risk of being unable to establish the given difference in treatments (disprove the null hypothesis). Therefore, in order to minimize the situation of ‘non-assessable’ patients solely due to missing data, we have incorporated the following measures.

1. Measures to minimize losses during follow-up: we have introduced a supporter system, usually a member of the patient’s family, to ensure full adhesion to the trial procedures by the patient and his/her support network. All trial sites have experience of observational studies or trials with long patient follow-up. We have also put into place a highly active patient tracking system with dedicated field workers who are notified when a patient misses a visit. We are fortifying this tracking system by building a further central level of quality control by the sponsor of the trial. Despite our best efforts we will inevitably lose patients, such as those migrating to neighboring countries in search for work.

2. Measures to minimize unavailability of bacteriological results for each patient at each visit. During the treatment phase two sputum samples were collected per patient and per visit at regular time points and cultured. Similarly, during the follow up phase, two sputum samples were collected per patient at regular time points and cultured (Table 3). Additional sputum samples are collected in cases of suspected relapse when the patient either presents with clinical symptoms of TB or has a positive sputum smear. This should minimize the risk of unavailable culture results.

### Open-label design

The trial is open-label (non-blinded) with respect to clinic staff reviewing patients at scheduled and unscheduled visits and the patients themselves. Laboratory technicians are blinded to the treatment regimen the patient is receiving. Double-blinding is favored in many trial situations; however it was felt that, with this trial design of a treatment shortening regimen, a non-blinded design was preferable. A patient’s knowledge that they are receiving a 4-month regimen may result in better adherence which arguably may not occur in a blinded design. This, to our view, is one of the most important aspect to capture when assessing a treatment shortening regimen. Nevertheless, one disadvantage of this design is that clinic staff are not blinded to treatment arm and may make different clinical decisions in caring for patients depending on the regimen a patient is receiving. For example, clinic staff may more readily retreat a patient, following the end of treatment if they were receiving the 4-month regimen which would penalize the test regimen arm. In light of this, our endpoints are based on mycobacteriological results (smear or culture). When mycobacteriological evidence is missing, endpoint classification has been defined and is discussed previously.

### Sample size determination (initial and revised calculations)

All sample size calculations were based on a power of 80% and a one-sided significance level of 2.5%. The initial sample size assumptions, presented in Table 6, were based on the outcome of relapses by 24 months following the end of treatment. Due to the revision of the primary outcome, following discussions with regulatory authorities, the sample size calculation also had to be revised. Revised assumptions had to take into consideration the composite ‘unfavourable’ outcome of treatment failure, recurrence or other ‘unfavourable’ scenarios, as listed in Table 4 by 24 months following the end of treatment. We used summarized internal blinded trial data from the interim analysis performed in May 2008 at the request of the DMC, to inform revised sample size assumptions (Table 6). We estimated the proportion with the revised unfavorable outcome by 24 months to be 20%. To account for the revised endpoint, margin of non-inferiority (delta) was increased to 6% (from 3%). Finally, losses to follow up (LTFU) were decreased to 15% (from 25%) since, patients who have died or been lost during treatment, and would have been considered LTFU for the original calculation, are considered as unfavorable for the revised outcome. Under revised assumptions we estimate 697 patients per arm (1,394 patients overall). Allowing for non-assessable patients the MITT analysis requires 1,640 patients overall.

**Table 6:** Original and revised sample size substantiation

	**Original calculation**	**Revised calculation**	
Outcome	Relapses at 24 months after end of treatment	‘Unfavourable’ events at 24 months after end of treatment (MITT^a^)	‘Unfavourable’ events 24 months after end of treatment (PP^b^)
Events in control arm (%)	5%	**20%**	**14%**
1-sided significance level	2.5%	2.5%	2.5%
Power	80%	80%	80%
Non-inferiority margin δ	3%	**6%**	**6%**
Patients overall (*n*)	1,656	1,394	1,050
Adjustment for LTFU^c^	20%	**15%**^d^	**27%**^d^
Patients after adjustment for LTFU (*n*)^d^	**2,070**	**1,640**	**1,438**

^a^Modified intent to treat.

^b^Per protocol.

^c^Lost to follow-up.

^d^In the revised sample size calculation, patients LTFU are re-classified as unfavorable or non-assessable. Therefore LTFU for the revised calculation refers to the adjustment made on the percentage of patients non-assessable.

However, since the per-protocol (PP) population analysis in non-inferiority studies is equally important to the MITT, we need to ensure sample size gives adequate power for the PP analysis as well. Deaths, losses, and withdrawals due to serious adverse events during treatment (estimated 6%) will most probably not satisfy the PP adherence criteria, as well as genuine non-adherers (estimated 6%) not included in the above categories. Based on these estimates, drawn from patient management logs, a further 12% of patients satisfying the MITT will be excluded from the PP analysis (Table 6). Furthermore, we estimate the proportion of patients with unfavorable outcome will be reduced to approximately 14% since deaths, losses, and withdrawals during treatment (unfavorable outcomes) are most probably excluded from this population analysis. For a power of 80% and delta of 6% we would require a total of 1,050 (525 per arm). Adjusting for 27% (15% + 12%) of patients excluded the PP requires 1,438 patients.

The total 1,836 patients recruited give OFLOTUB enough power for both the MITT and PP analyses. An independent review by a statistician to confirm the revision of sample size was undertaken and the DMC was kept informed throughout the process.

## Conclusion

We have presented in this manuscript key questions we were required to answer while designing OFLOTUB, and our reasoning for the decisions we made. There is no single answer for any of these design questions in the context of phase III TB drug trials and this is demonstrated by current consultations within regulatory agencies. When shortening TB treatment, we are advocating for a non-inferiority, non-blinded design, with a composite unfavorable endpoint assessed 12 months post treatment completion, and added trial procedures specifically aiming to: (1) minimize endpoint unavailability; and (2) distinguish between relapse and re-infection.

With our paper we are adding to the sparse published literature on the design of pivotal TB drug trials and hope to inform decision making for such future trials.

## Competing interests

The authors declare that they have no competing interests.

## Authors’ contributions

CSCM led the writing and co-wrote the paper. CS co-wrote the paper. MG, JH, OL, MBL, DM, CP, FP, JO, PO, RR, and CL revised the paper. KF supervised the writing of the paper. All authors read and approved the final manuscript.
